# Increased longitudinal contractility and diastolic function at rest in well-trained amateur Marathon runners: a speckle tracking echocardiography study

**DOI:** 10.1186/1476-7120-12-11

**Published:** 2014-02-26

**Authors:** Sebastian Schattke, Yan Xing, Jürgen Lock, Lars Brechtel, Sabrina Schroeckh, Sebastian Spethmann, Gert Baumann, Adrian C Borges, Fabian Knebel

**Affiliations:** 1 Medizinische Klinik für Kardiologie und Angiologie, Campus Mitte, Charité —Universitätsmedizin Berlin, Charitéplatz 1, D – 10117 Berlin, Germany; 2 Klinik für Innere Medizin I – Kardiologie, Helios Klinikum Emil von Behring, Berlin, Germany; 3 Department of Cardiology, East Hospital, Tongji University, Shanghai, China; 4 SCC Running, Berlin, Germany; 5 Department of Sports Medicine, Humboldt University of Berlin, Berlin, Germany

## Abstract

**Background:**

Regular physical activity reduces cardiovascular risk. There is concern that Marathon running might acutely damage the heart. It is unknown to what extent intensive physical endurance activity influences the cardiac mechanics at resting condition.

**Methods:**

Eighty-four amateur marathon runners (43 women and 41 men) from Berlin-Brandenburg area who had completed at least one marathon previously underwent clinical examination and echocardiography at least 10 days before the Berlin Marathon at rest. Standard transthoracic echocardiography and 2D strain and strain rate analysis were performed. The 2D Strain and strain rate values were compared to previous published data of healthy untrained individuals.

**Results:**

The average global longitudinal peak systolic strain of the left ventricle was -23 +/- 2% with peak systolic strain rate -1.39 +/- 0.21/s, early diastolic strain rate 2.0 +/- 0.40/s and late diastolic strain rate 1.21 +/- 0.31/s. These values are significantly higher compared to the previous published values of normal age-adjusted individuals. In addition, no age-related decline of longitudinal contractility in well-trained athletes was observed.

**Conclusions:**

There is increased overall longitudinal myocardial contractility at rest in experienced endurance athletes compared to the published normal values in the literature indicating a preserved and even supra-normal contractility in the athletes. There is no age dependent decline of the longitudinal 2D Strain values. This underlines the beneficial effects of regular physical exercise even in advanced age.

## Background

There is an increasing participation in long-distance running events; there is clear evidence that regular physical activity reduces cardiovascular risk [1-3]. Still, it is unknown if the intensity of physical activity and the beneficial effects correlate linearly and whether Marathon running is dangerous for the cardiovascular system [4]. However, some studies on Marathon running raised concerns of acute and sustained myocardial injury of the left and the right ventricle assessed by cardiac biomarkers and echocardiography [5,6]. Other studies could not confirm exercise-induced myocardial damage [7,8]. Several studies have been published with the aim of evaluation of left and right ventricular function in athletes with different forms of training including endurance recently. These studies were focused only on young and elite athletes [9,10]. Up to date, there are rare data about the cardiac mechanics at resting condition of well endurance-trained amateur athletes with a broad range of age.

Two-dimensional strain is a new Doppler independent approach for calculation of strain, strain rate, tissue velocity, and displacement. It is based on speckle tracking analysis in B-Mode and therefore less angle-dependent than Doppler-derived strain analyses [11]. This novel echocardiographic approach has improved the assessment of myocardial regional and global systolic and diastolic function. For routine application of myocardial two-dimensional strain in clinical practice normal ranges have been defined and published recently [12,13]. It has been shown, that global 2D strain and strain rate are independent to age, gender, heart rate and systolic blood pressure [12]. While TDI velocities (s’ e’ a’) are age dependent in a large cohort of healthy (non marathon runners) volunteers [14]. Furthermore it is known that diastolic left ventricular dysfunction has a higher prevalence in elderly and it leads to age-related normal values of mitral inflow velocities and time intervals [15,16]. In contrast to this observation a small study described that left ventricular compliance can be preserved in elderly by regular endurance training [17].

The influence of long standing endurance training on the normal resting values of longitudinal strain has not been examined yet in a larger cohort.

This study aims to describe the normal resting values of systolic and diastolic left ventricular function assessed by speckle tracking in amateur marathon runners, to compare the age dependence in this cohort and to compare the values with recently published normal values in non athletics.

## Methods

### Study population

The organizers of the (2006 and 2007) Berlin Marathon invited local registered amateur runners by e-mail to participate in our study. Runners from Berlin-Brandenburg area who had completed at least one marathon previously were included into this study. All participants underwent clinical examination and echocardiography at least 10 days before the marathon at rest. Exclusion criteria were known cardiovascular disorders (atrial fibrillation, pacemaker, bypass surgery, prosthetic valves or congenital heart disease), signs and symptoms of coronary artery disease, recent pathologic stress test, hypertension and diabetes. All participants had a normal standard echocardiography with no signs of cardiac pathology. In addition, an ECG and a blood sample were taken from every participant. The study protocol was approved by the ethics committee of the Charité University Hospital and conducted according to the Helsinki Declaration. Written informed consent was obtained from each participant. Twenty two healthy non-athlete adults served as controls.

### Echocardiography

**Figure 1:**
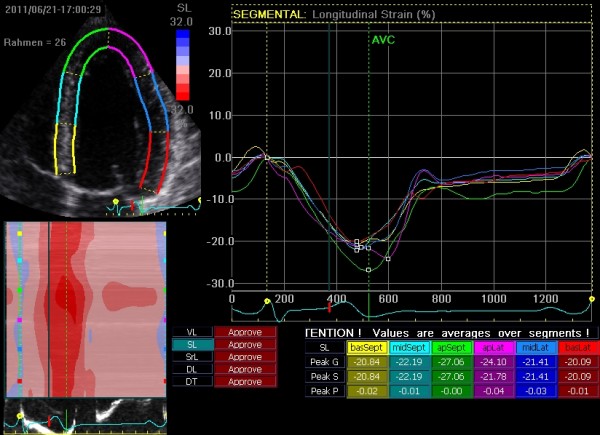
2D-Strain analysis of the left ventricle in the apical two chamber view with average values for peak systolic strain (Peak S) peak global strain (Peak G) and peak positive strain (Peak P) over each segment.

Standard transthoracic echocardiography was performed according to the guidelines of the American Society of Echocardiography (ASE) and the European Association of Cardiovascular Imaging (EACVI) on Vivid 7 Dimension (GE Vingmed, Horton, Norway, M3S 1.5-4.0 MHz transducer) [18]. Segmental Color Doppler recordings were performed to exclude valvular dysfunction. Real-time 2D ultrasound data from the left ventricle at the three apical views with a frame rate greater than 50 frames per second (fps) were stored digitally for offline 2D strain and strain rate analysis (EchoPac PC, GE Vingmed, Horton Norway) (Figure 1). Aortic valve opening and closure was set manually by the opening and closing artefact using a transaortic continuous-wave Doppler signal in order to define end systole for 2D strain analysis. For segmental longitudinal strain and strain rate analysis an 18 segment- model of the LV were used by dividing each LV wall into 3 segments. The region of interest (ROI) was corrected manually in order to analyze the myocardium and to exclude the pericardium. For each segment the quality of speckle tracking was analyzed automatically. Segments with poor tracking were excluded for further measurements. To achieve average 2D peak systolic strain the mean of every wall and then the average of all walls was calculated. Strain rate was measured at the most negative point of SR during the ejection phase. The early (E’) and late (A’) diastolic strain rate was measured as a marker of the diastolic function in this cohort.

### Statistics

Results are expressed as mean value ± standard deviation (SD). Outliers and/or skew distributions differences in interesting groups of individuals were analyzed using nonparametric statistical tests (Mann–Whitney U-test). Spearman’s correlation coefficient was calculated in order to test for variable dependencies of 2D strain parameters. Statistical analyses were performed using SPSS for Windows (Release 19.0, Copyright ® SPSS Inc.). The comparison of our results and the published values in the literature was performed by the t-test. Significance was assessed at the p < 0.05 level.

The intra- and interobserver variability was measured on different days by three different cardiologists blinded to the results of the other cardiologists. 10 healthy individuals were examined. We calculated the mean longitudinal 2D strain in each segment. We have then calculated the delta of each individual measurement and the mean. Finally the average of the deltas was calculated to measure the intra and inter observer variability.

## Results

### Subject characteristics

Eighty-four marathon runners, 43 women and 41 men participated in this study (mean age 50 ± 11 years). All participants had a normal ECG and blood test including NTproBNP in the normal age and gender-adjusted range according to Hess [19]. The normal NTproBNP levels underline the echocardiography results of cardiac healthy population in our study. The systolic and diastolic blood pressure as well as heart rate and weekly average training differ not significantly between female and male amateur runners. The male runners had a higher running experience explained in a higher amount of previous marathons. The female runners were slightly younger and there was a significant difference in body mass index between the groups. The baseline characteristics, training level and running experience are shown in Table 1.

**Table 1:** Baseline characteristics, training level and running experience

	**All runners**	**Male**	**Female**	***P***
Number	84	41	43	
Age, years	50 ± 11 (22–69)	52 ± 14 (22–69)	48 ± 9 (22–69)	0.044
Body mass index, kg/m^2^	22 ± 2	23 ± 2	21 ± 2	< 0.001
Blood pressure, mmHg				
*Systolic*	126 ± 14	130 ± 14	124 ± 15	0.052
*Diastolic*	81 ± 8	83 ± 9	80 ± 8	0.067
Baseline heart rate, 1/min	63 ± 9	62 ± 8	63 ± 9	0.662
Weekly average training, km	54 ± 19	55 ± 23	53 ± 16	0.547
Previous marathons (n)	14 ± 25	21 ± 33	7 ± 10	< 0.001
NT-proBNP [pg/ml]	91 ± 73	68 ± 56	113 ± 81	0.001

### Feasibility of 2D strain measurements

1450 LV wall segments (96%) of a total of 1512 segments had sufficient speckle tracking quality and could be analyzed. The best tracking was detectable in the septum and in the basal and medial segments of all LV walls. The worst quality was seen in the anterior wall and in the apex. The number of analyzed segments is summarized in Table 2.

**Table 2:** Feasibility of 2D-strain, number of analyzed LV-segments

**LV segments**	**Basal**	**Medial**	**Apical**	**All levels**
Septal	84	84	82	250
Lateral	82	80	77	239
Inferior	82	82	74	238
Anterior	81	80	74	235
Posterior	83	84	78	245
Anteroseptal	83	84	76	243
All walls	495	494	461	1450

### 2D strain values

**Figure 2:**
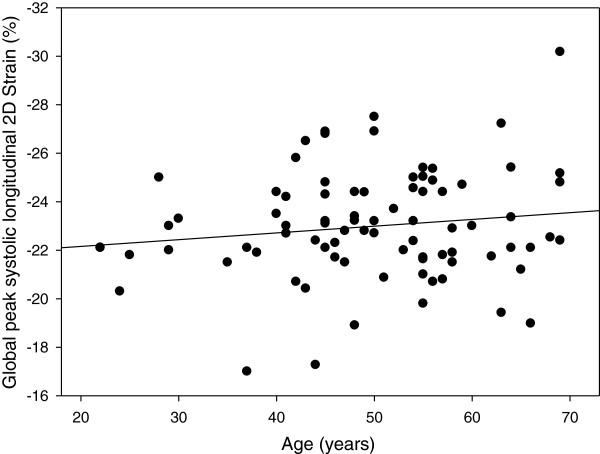
Age dependence of longitudinal 2D strain (Spearman correlation coefficient 0.073, p = 0.51).

Table 3 shows the mean values of peak systolic 2D strain of all LV segments. The average peak systolic strain in the basal segments was significantly lower than in the mid-ventricular segment in *all LV walls except posterior (P = 0.751) and inferior (P = 0.282)*. The average peak systolic strain in the mid-ventricular segments was significantly lower than the values of the apical segment in all LV walls. The average global longitudinal peak systolic strain of the left ventricle was -23 ± 2% with peak systolic strain rate -1.39 ± 0.21/s, early diastolic strain rate 2.0 ± 0.40/s and late diastolic strain rate 1.21 ± 0.31/s (Table 4). The global longitudinal peak systolic strain of the left ventricle differed significantly between female and male amateur runners with lower systolic deformation indices in male runner group. This phenomenon is due to significantly different average strain values in the apical long axis (posterior and anteroseptal wall) and two chamber (inferior and anterior wall) view, while in the four chamber view no significant difference were seen between the groups (Table 5).There is no correlation between age and average global longitudinal 2D Strain the whole study group (Figure 2).

**Table 3:** Mean segmental longitudinal peak systolic strain values in %

	**All levels**	**Apical**	**Mid**	**Basal**
All walls	-23.0 ± 2.2	-25.6 ± 4.9	-22.6 ± 3.5	-20.9 ± 4.1
Anterior	-22.8 ± 4.4	-24.6 ± 4.8	-22.6 ± 3.9	-21.5 ± 4.0
Anteroseptal	-22.6 ± 5.0	-25.7 ± 5.5	-23.0 ± 3.0	-19.2 ± 4.1
Inferior	-23.8 ± 4.5	-25.2 ± 5.1	-23.5 ± 4.1	-23.0 ± 4.0
Lateral	-23.4 ± 4.3	-26.5 ± 4.5	-22.6 ± 3.1	-21.2 ± 3.2
Posterior	-23.2 ± 4.3	-24.6 ± 4.9	-22.6 ± 3.3	-22.5 ± 4.3
Septal	-22.1 ± 5.0	-27.1 ± 4.1	-21.2 ± 3.0	-18.1 ± 2.8

**Table 4:** Comparison of our results with published normal values in healthy individuals

**Parameter**	**Peak systolic strain(%)**	**Peak systolic SR(1/s)**	**Early diastolic SR(1/s)**	**Late diastolic SR(1/s)**
**Athlete** (n = 84)	-23.0 ± 2.23	-1.39 ± 0.21	2.00 ± 0.40	1.21 ± 0.31
**Control Marwick** (n = 192)	-18.6 ± 0.1	-1.1 ± 0.01	1.55 ± 0.01	1.02 ± 0.01
p	< 0.001	< 0.001	< 0.001	< 0.001
**Control HUNT study** (n = 1266)	-16.7 ± 4.1	-1.03 ± 0.27	no data	no data
p	< 0.001	< 0.001	-	-

**Table 5:** Global peak systolic strain values (%) compared by sex

**Global peak systolic strain**	**Female**	**Male**	**P**
APLAX	-23.3 (± 2.5)	-22.1 (± 2.6)	0.011
2CH	-24.1 (± 2.7)	-22.1 (± 2.6)	< 0.001
4CH	-23.5 (± 2.7)	-22.7 (± 2.8)	0.126
Average left ventricular strain	-23.6 (± 2.1)	-22.3 (± 2.2)	0.001

APLAX, apical long axis view; 2CH, apical two chamber view; 4CH, apical four chamber view.

The interobserver variability for longitudinal 2D strain measurements was 5,0% and the intraobserver variability was 8,4%.

### Comparison with controls

Table 4 shows the results of our cohort compared to previous published normal 2D strain values in healthy individuals. For comparison we used the data published by Marwick et al. and from the large HUNT Study [12,13]. The global left ventricular longitudinal peak systolic strain and systolic strain rate of our cohort are significantly higher compared to the previous published values of normal individuals. The same phenomenon was observable for the early and late diastolic strain rate values, E’ and A’ (Table 4). The average longitudinal 2D strain at rest of the healthy non-athlete adult controls (n = 22) was -21.1 ± 2.5% (p < 0.001, compared with athletes).

## Discussion

Our study is the first that has examined normal values of left ventricular longitudinal 2D strain for amateur endurance athletes. The main result of our study is that well-trained athletes have a significantly higher average strain values compared to previously published data of untrained healthy controls. This indicates that resting systolic and diastolic myocardial performance is better in well-trained individuals.

The invasive study by Arbab-Zadeh has shown an increased stroke volume in endurance master athletes at rest. They could also show a better contractility in the master athletes compared to an age-matched control group [17]. We could show an increased contractility at rest also in amateur runners compared to untrained healthy individuals by a non-invasive echo method.

Two large TDI and speckle tracking studies have shown an age-dependant decrease of myocardial deformation in a normal healthy population [13,14]. Our 2D strain data shows no age-related decline of longitudinal contractility in well-trained athletes. Furthermore, the values of the elderly runners are supra-normal compared to the data of Dalen [12] and Marwick [13]. This indicates that in higher ages systolic myocardial performance seems to be preserved in well trained persons; this underlines previous observations of Arbab-Zadeh [17], that elderly master athletes have a myocardial function comparable to young sedentary individuals. One can speculate, that the frequently recognized physiological age-related changes might alternatively be explained as the negative consequences of a sedentary lifestyle.

Not only systolic function, but also diastolic function (as measured by E’ and A’ Strain rates) was higher in the well-trained athletes compared to the published controls [12]. This underlines the beneficial effects of regular exercise and is supported by the work of Arbab-Zadeh, which has shown in an invasive study that persons with regular endurance training have lower LV filling pressures and a better left ventricular compliance [17].

Which mechanism could potentially explain these differences? It could be due to different ages and heart rates of the controls and the athletes. However, the heart rate and the age of the control patients are comparable. In our study the mean heart rate was 63 ± 9 bpm, in the HUNT study 66 ± 10/63 ± 10 (male/female), in the study published by Marwick et al., the mean heart rate is not mentioned. The age of our athletes (50 ± 11 years) and the HUNT study population study (48 ± 14 male/51 ±14 female) and the Marwick study (51 ± 12) is comparable [12,13].

As heart rate and age differences do not seem to explain the differences, we speculate based on our observations, that the well trained amateur runners seem to have an increased resting myocardial performance in systole and diastole.

Apart from established cardiovascular risk factors (smoking, hypertension, hyperlipidaemia, diabetes, body mass index) recent epidemiological studies have confirmed that regular physical exercise contributes to reduce mortality [20]. The observed increased myocardial performance in our cohort is possibly one explanation for the beneficial effect of regular exercise.

The correlation between contractility and mortality has been demonstrated previously in patients with heart failure (with reduced ejection fraction and preserved systolic function). Various studies demonstrated a reduced prognosis with reduced contractility [21], and a better prognosis after sustained improvement of contractility [22,23]. Several factors have been discussed to be important factors for better prognosis in athletes: autonomic status, resting heart rate, heart rate recovery after exercise, heart rate variability and parasympathetic activity. Cardiac contractility might be one additional aspect in this context [24].

However, this study did not have the aim to demonstrate whether increased contractility is reason or major effect or sign for good prognosis.

## Conclusion

This study establishes normal values for longitudinal 2D Strain, Strain Rate and diastolic Strain Rate in a large cohort of healthy well-trained amateur endurance athletes. There is an increased overall longitudinal myocardial contractility at rest compared to the published normal values in the literature indicating a preserved and even supra-normal contractility. We also found significantly better values for diastolic function in our cohort compared to the previous published values of normal individuals indicating a better left ventricular compliance.

There is no age dependent decline of the longitudinal 2D Strain and diastolic values. This underlines the beneficial effects of regular physical exercise even in advanced age.

### Limitations

There is no age and gender matched control group in this study. The comparisons were drawn to historic controls in the literature. Secondly, we have only analyzed longitudinal 2D Strain and do not have invasive data. Our study did not examine age and gender differences.

## Competing interests

The authors declare that they have no competing interests.

## Authors’ contributions

SS, Conception and design of the study, analysis and interpretation of echocardiographic data, drafting and revision of the manuscript. YX Conception and design of the study, analysis and interpretation of echocardiographic data. JL, LB and SS, Conception and design of the study. SS, Conception and design of the study, revision of the manuscript. GB, Conception and design of the study. ACB, Conception and design of the study, revision of the manuscript. FK, Conception and design of the study, analysis and interpretation of echocardiographic data, drafting and revision of the manuscript. All authors read and approved the final manuscript.
